# Analgesic and Anti-Inflammatory Activities of Methanol Extract of *Ficus pumila* L. in Mice

**DOI:** 10.1155/2012/340141

**Published:** 2012-05-14

**Authors:** Chi-Ren Liao, Chun-Pin Kao, Wen-Huang Peng, Yuan-Shiun Chang, Shang-Chih Lai, Yu-Ling Ho

**Affiliations:** ^1^School of Chinese Pharmaceutical Sciences and Chinese Medicine Resources, College of Pharmacy, China Medical University, Taichung 404, Taiwan; ^2^Department of Nursing, Hsin Sheng College of Medical Care and Management, Taoyuan 325, Taiwan; ^3^Department of Health and Nutrition Biotechnology, Asia University, Taichung 413, Taiwan; ^4^Department of Nursing, Hungkuang University, Sha Lu, Taichung 433, Taiwan

## Abstract

This study investigated possible analgesic and anti-inflammatory mechanisms of the methanol extract of *Ficus pumila* (FP_MeOH_). Analgesic effects were evaluated in two models including acetic acid-induced writhing response and formalin-induced paw licking. The results showed FP_MeOH_ decreased writhing response in the acetic acid assay and licking time in the formalin test. The anti-inflammatory effect was evaluated by *λ*-carrageenan-induced mouse paw edema and histopathological analyses. FP_MeOH_ significantly decreased the volume of paw edema induced by *λ*-carrageenan. Histopathologically, FP_MeOH_ abated the level of tissue destruction and swelling of the edema paws. This study indicated anti-inflammatory mechanism of FP_MeOH_ may be due to declined levels of NO and MDA in the edema paw through increasing the activities of SOD, GPx, and GRd in the liver. Additionally, FP_MeOH_ also decreased the level of inflammatory mediators such as IL-1*β*, TNF-*α*, and COX-2. HPLC fingerprint was established and the contents of three active ingredients, rutin, luteolin, and apigenin, were quantitatively determined. This study provided evidence for the classical treatment of *Ficus pumila* in inflammatory diseases.

## 1. Introduction

Inflammatory reaction, typically characterized by redness, swelling, heat, and pain, is one of the most important host defense mechanisms against invading pathogens. However, persistent or overinflammation leads to tissue damage and possibly failure of organs. Proinflammatory cytokines (e.g., TNF-*α*, IL-1*β*, and IL-6) are produced in large quantities by activated macrophages/monocytes that stimulate cellular responses via increasing prostaglandins (PGs) and reactive oxygen species (ROS). Additionally, lipid peroxidation (malondialdehyde, MDA) is produced by free radicals attacking the cell membranes. Thus, inflammatory effect can lead to the accumulation of MDA [1].

*Ficus pumila*, a creeping vine like fig plant, is native to South China and Malaysia. Several studies have been performed on the composition of *Ficus pumila*, and a number of compounds have been identified such as apigenin, luteolin [2], rutin, genistein, hesperidin, astragalin, isoquercitrin, and chrysin [3]. Dried stems and leaves of *Ficus pumila* have been folklorically used in the treatment of rheumatoid arthritis, edema, tonic medicament, throat pain, and postpartum abdominal pain [4]. However, no research has been investigated on the analgesic and anti-inflammatory mechanisms of *Ficus pumila* yet.

In this study, we investigated the analgesic and anti-inflammatory activities of the methanol extract of *Ficus pumila* (FP_MeOH_). The analgesic activity was evaluated by acetic acid-induced writhing response and formalin test. Anti-inflammatory activity was determined by using **λ**-carrageenan-induced mouse paw edema model and histopathological analysis. In order to evaluate the mechanism of anti-inflammatory effect, we also analyzed TNF-*α*, IL-1*β*, COX-2, MDA, and NO levels in the edema tissue, as well as antioxidant enzyme activities of SOD (superoxidase dismutase), GPx (glutathione peroxidase), and GRd (glutathione reductase) in the liver.

Many studies have indicated that flavonoids in herbs possess anti-inflammatory activities via scavenging ROS and reducing proinflammatory cytokines, such as rutin [5], luteolin [6], and apigenin [7]. These three ingredients have also been isolated from *Ficus pumila* in previous studies [2, 3]. In the phytochemical part of this study, not only did we reconfirm the presence of these three compounds in FP_MeOH_ by establishing its fingerprint chromatogram, but also the contents of these active ingredients were quantitatively determined.

## 2. Materials and Methods

### 2.1. Chemicals and Drugs

**λ**-carrageenan, indomethacin, and Griess reagent were purchased from Uni-Onward (distributor of Sigma-Aldrich Chemical Co. in Taipei, Taiwan). Formalin was purchased from Nihon Shiyaku Industry Ltd. (Taipei, Taiwan). SOD, GPx, GRd, and MDA assay kits were purchased from Eugene Chen Co. Ltd. (distributor of Randox Laboratory Ltd. in Taipei, Taiwan). IL-1*β*, IL-6, COX-2, and TNF-*α* were obtained from Blossom Biotechnologies, Inc. (agency of Assay Designs Inc. in Taipei, Taiwan). LC grade acetonitrile was purchased from the branch company of Merck in Taipei, Taiwan. All other reagents used were of analytical grade.

### 2.2. Plant Material

*Ficus pumila* L. was collected from the herbal garden of China Medical University, Taichung, Taiwan, as described in Flora of Taiwan [8]. A plant specimen has been deposited in the School of Chinese Pharmaceutical Sciences and Chinese Medicine Resources. Dried stems and leaves (1.0 kg) of *Ficus pumila *were sliced into small pieces, soaked with 10 liters of methanol at room temperature for three days. After passing through filter paper, the filtrate was concentrated under reduced pressure to dryness. The above steps were repeated two additional times, and a total of 123.1 g of dry crude extract (yield ratio 12.31%) was produced. The dried crude extract was dissolved in 0.5% CMC (carboxymethyl cellulose) solution into three concentrations (100 mg/mL, 50 mg/mL, and 10 mg/mL) prior to pharmacological testing. Equal volume of 0.1 mL (extract of different concentrations)/10 g B.W. was given to each mouse in all subsequent animal experiments.

### 2.3. Chromatographic Analysis of FP_MeOH_

 The HPLC system consisted of a Waters 2695 Alliance LC with 996 PDA. Chromatographic separation was performed on X-Bridge RP18 (25 cm × 4.6 mm I.D., 5 *μ*m) with an injection volume of 10 *μ*L. The mobile phase consisted of a mixture of 0.1% formic acid (A) and acetonitrile (B) using a gradient elution. The gradient program was set as follows: 0–15 min, 20% B, 15–30 min, 40% B, 30–45 min, 60% B. The flow rate was set at 0.8 mL/min and the detection wavelength was set to 255 nm. The above conditions were used in both HPLC assay and HPLC fingerprint of FP_MeOH_.

The contents of rutin, apigenin, and luteolin of FP_MeOH_ were qualified and quantified in the HPLC assay. In qualitative analyses, comparisons were made with the retention time and maximum absorption of the standards. In quantitative analyses, comparisons were made with peak areas under the standard curves.

### 2.4. Experimental Animals

 Male ICR mice aged between 5-6 weeks and weighing between 20–25 g were obtained from BioLASCO Taiwan Co. Ltd. and were kept in the animal center of China Medical University at a controlled temperature of 22 ± 1°C, relative humidity 55 ± 5%, and with 12 h light/12 h dark cycles for 1 week before the experiment. Animals were provided with rodent diet and clean water *ad libitum*. The experimental protocol was approved by the Committee on Animal Research, China Medical University. Animals were sacrificed by decapitation under ether anesthesia. All studies were conducted in accordance with the National Institutes of Health (NIH) Guide for the Care and Use of Laboratory Animals. All tests were conducted under the guidelines of the International Association for the Study of Pain [9].

### 2.5. Acute Toxicity Study

 The acute toxicology test in mice was carried out according to the method of Liao et al. [10]. Male ICR mice were randomly divided into three groups (10 mice per group). Three groups of mice were administered orally with three concentrations of FP_MeOH_ (2.5 g, 5 g, and 10 g/kg), respectively. The experimental mice were kept under regular observation for 14 days for any mortality or behavioral changes.

### 2.6. Acetic Acid-Induced Writhing Response

The writhing test in mice was carried out according to the method of Koster et al. [11]. Five randomly-selected groups of mice were orally administered with solvent control (0.5% CMC), positive control (indomethacin at 10 mg/kg) or three different doses of FP_MeOH_ (0.1, 0.5, and 1.0 g/kg) 60 min prior to the chemical stimulus. The number of muscular contractions was counted 5 min after the injection of 1% acetic acid (v/v, 0.1 mL/10 g body weight, i.p.). The data collected represented the total number of writhes observed in a duration of 10 minutes (5–15 min after the injection).

### 2.7. Formalin Test

The formalin test was conducted based on the method of Tjølsen et al. [12]. Twenty microliters of 5% formalin in saline was injected subcutaneously into the right hind paw of each mouse. The time (in seconds) spent on licking and biting of the injected paw was recorded in both the early phase (0–5 min) and late phase (20–30 min) after the formalin injection. Solvent control (0.5% CMC), positive control (indomethacin at 10 mg/kg), or three different doses of FP_MeOH_ (0.1, 0.5 and 1.0 g/kg) was orally administered to the animals 60 min prior to the formalin injection.

### 2.8. *λ*-Carrageenan-Induced Mouse Paw Edema

 The test was conducted according to the method of Vinegar et al. [13]. The basal volume of right hind paw was determined before the administration of any drug. Fifty microliters of 1%  *λ*-carrageenan suspended in saline was injected into the plantar side of right hind paw, and the paw volume was measured at the 1st, 2nd, 3rd, 4th, and 5th h after the injection using a plethysmometer. The degree of swelling was evaluated by the delta volume (a-b), where “a” is the volume of right hind paw after the chemical treatment and “b” is the volume before the treatment. CMC (0.5%), indomethacin (10 mg/kg), and FP_MeOH_ (0.1, 0.5 and 1.0 g/kg) were administered orally 60 min after the **λ**-carrageenan injection. In the secondary experiment, another set of mice were orally administered with 0.5% CMC, indomethacin, or FP_MeOH_ 1 h after *λ*-carrageenan had been injected into their right hindpaws. The right hindpaws of the animals were surgically removed under anesthesia 2 h following treatments. The paw tissue was rinsed in ice-cold normal saline and immediately placed in cold normal saline four times its volume before homogenization at 4°C. The homogenate was centrifuged at 12,000 rpm for 5 min. The supernatant was obtained and stored at −20°C for the determinations of MDA, NO, TNF-*α*, IL-1*β*, and COX-2. Similarly, the whole liver tissue was rinsed in ice-cold normal saline and immediately placed in cold normal saline of equal volume before homogenization at 4°C. The homogenate was then centrifuged at 12,000 rpm for 5 min. The supernatant was obtained and stored at −20°C for later analyses of antioxidant enzyme (SOD, GPx, and GRd) activities.

### 2.9. Histological Analysis

 The mice were orally administered with 0.5% CMC, indomethacin, or FP_MeOH_ 1 h after *λ*-carrageenan had been injected into their right hindpaws. The right hindpaws of the animals were surgically removed under anesthesia 2 h after treatments. Tissue slices were fixed in 10% formalin for 3 days, decalcified overnight and embedded in paraffin and sectioned into 4 *μ*m tissue sections. Tissue sections were stained with hematoxylin and eosin (H&E stain) before being examined under a BX60 microscope (Olympus, Melville, NY) for pathological changes. Inflammatory reactions induced by *λ*-carrageenan, including paw swelling and enlarged cavities, were examined. The severity after FP_MeOH_ (0.1, 0.5 and 1.0 g/kg) and indomethacin (10 mg/kg) treatments was also examined. Images were captured with a Macrofire 599831 camera. The results were identified in the Animal Disease Diagnostic Center (ADDC), National Chung Hsing University, Taichung, Taiwan.

### 2.10. MDA Assay

 The production of MDA was induced by *λ*-carrageenan injection, and evaluated by the thiobarbituric acid reacting substance (TBARS) method [14]. Briefly, MDA reacted with TBARS at high temperature and formed a red-complex TBARS. The absorbance of TBARS was recorded at 532 nm.

### 2.11. NO Assay

NO was measured according to the method of Moshage et al. [15]. NO_3_^−^ was converted into NO_2_^−^ by nitrate reductase, NO_2_^−^ subsequently reacted with sulfanilic acid to produce diazonium ion and coupled with N-(1-naphthyl) ethylenediamine to form the chromophoric aero-derivative (purplish red) which could be recorded at 540 nm.

### 2.12. TNF-*α* and IL-1*β* Assay

 IL-1*β* was measured by an enzyme-linked immunosorbent assay kit (Blossom Biotechnologies Inc.) [16]. The capture antibody of IL-1*β* was added to each well of a 96-well plate overnight. Next day, a second set of biotinylated antibody was incubated with sample tissues or standard antigens in the plate before streptavidin was added. The color of the reaction converted from purple to yellow and was recorded at 450 nm. TNF-*α* was detected using the same method as IL-1*β*. Each sample was presented as pg/mg in TNF-*α* and IL-1*β* concentrations.

### 2.13. COX-2 Assay

The content of COX-2 was determined by measuring the peroxidase activity of PGHS (prostaglandin endoperoxide H_2_ synthase) [17]. Peroxidase activity of PGHS was determined by following the oxidation of N,N,N′,N′-tetramethyl-p-phenylenediamine (TMPD) at 37°C using arachidonate as the substrate. The increase in color was recorded at 590 nm.

### 2.14. Measurement of Antioxidant Enzymes

SOD was measured according to the method of Vani et al. [18]. Xanthine and xanthine oxidase (XOD) generated superoxide radicals reacted with 2-(4-iodophenyl)-3-(4-nitrophenol)-5-phenyl-tetrazolium chloride (I.N.T.) to form a red formazan dye, and the color was recorded at 540 nm. GPx was measured according to the method of Ceballos-Picot et al. by detecting the contents of GR and NADPH [19]. Oxidation of NADPH into NADP^+^ is accompanied by a decrease in absorbance recorded at 340 nm. GRd was measured according to the method of Ahmad and Holdsworth [20] which detects the decrease of glutathione (GSSG) in the presence of NADPH. NADPH oxidized into NADP^+^ would result in a decrease in absorbance recorded at 340 nm.

### 2.15. Statistical Analysis

All data of each group were expressed as mean ± SD (*n* = 8). Statistical analyses were performed with SPSS software and were carried out using one-way ANOVA followed by Scheffe's multiple range test.

## 3. Results

### 3.1. Chromatographic Analysis of FP_MeOH_

**Figure 1:**
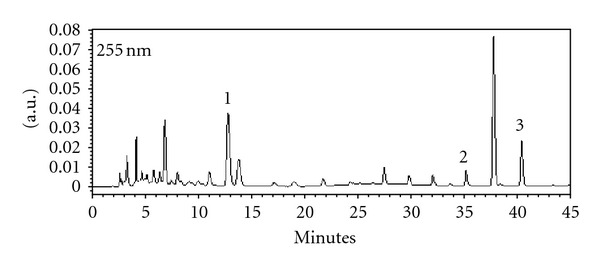
HPLC fingerprint of FP_MeOH_. The peaks represent 1. rutin (13.8 min), 2. luteolin (35.2 min), and 3. apigenin (40.4 min). The contents of rutin, apigenin, and luteolin in FP_MeOH_ were 24.41 mg/g, 14.11 mg/g, and 2.72 mg/g, respectively.

HPLC fingerprint profile was established for FP_MeOH_ (Figure 1). Three flavonoid components were identified as rutin, luteolin, and apigenin with retention times of 13.8 min, 35.2 min, and 40.4 min, respectively. The maximum absorbance was 255 nm, and the relative amounts for each gram of crude extract were in the order of rutin (24.41 mg), apigenin (14.11 mg), and luteolin (2.72 mg).

### 3.2. Acute Toxicity Study

Acute toxicity of FP_MeOH_ was evaluated in mice at the doses of 2.5, 5 and 10 g/kg. After 14 days of oral administration, FP_MeOH_ did not cause any behavioral changes, and no mortality was observed. Therefore, the LD_50_ value of FP_MeOH_ was concluded to be greater than 10 g/kg in mice, indicating it was practically not acutely toxic.

### 3.3. Acetic Acid-Induced Writhing Response

**Figure 2:**
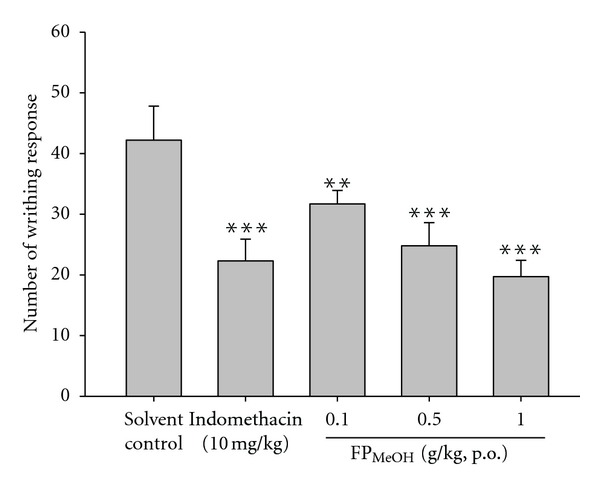
Analgesic effects of FP_MeOH_ and indomethacin on acetic acid-induced writhing response in mice. Each value represents mean ± SD (*n* = 8). The number of abdominal writhes was counted over the time period of 5–15 min after acetic acid injection. **P < 0.01 and ***P < 0.001 as compared to the solvent control group (one-way ANOVA followed by Scheffe's multiple range test).

Figure 2 shows acetic acid-induced writhing responses in mice which serve as an indication of analgesic activities of FP_MeOH_. Intraperitoneal injection of acetic acid produced 42.2 ± 5.6 writhes in the solvent control group. The writhing response was significantly reduced by pretreatments with FP_MeOH_ (0.1, 0.5 and 1 g/kg) and indomethacin (10 mg/kg).

### 3.4. Formalin Test

**Figure 3:**
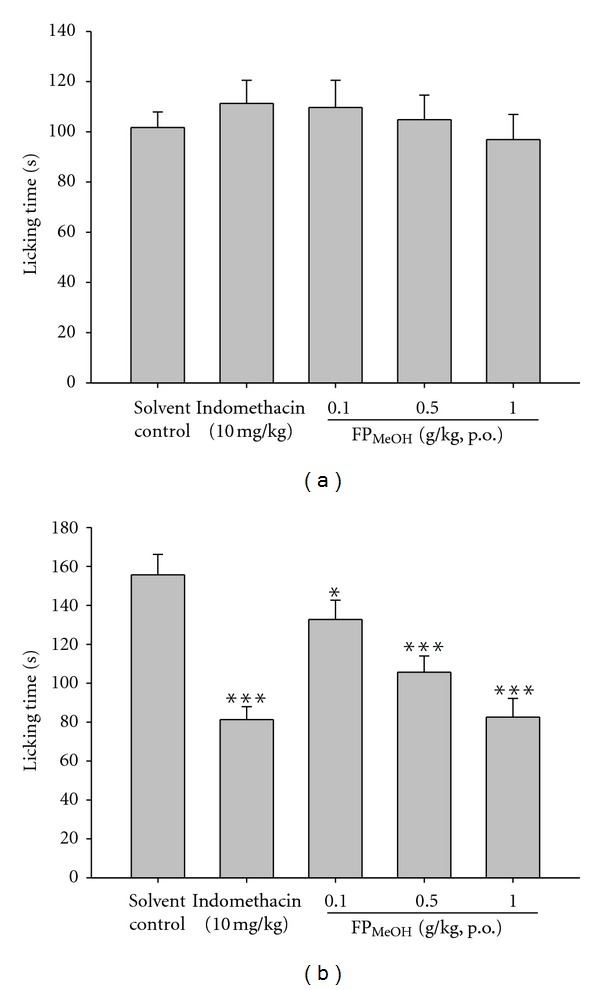
Effects of FP_MeOH_ and indomethacin on (a) early and (b) late phases of formalin test in mice. Each value represents mean ± SD (*n* = 8). The time spent on licking and biting the injected paws was recorded in the time periods of 0–5 min (early phase) and 20–30 min (late phase) as indicators of pain. *P < 0.05 and ***P < 0.001 as compared to the solvent control group (one-way ANOVA followed by Scheffe's multiple range test).

 In the early phase, FP_MeOH_- and indomethacin-treated groups did not show any significant changes as compared to the solvent control group (Figure 3(a)). In the second phase, subcutaneous injection of formalin induced licking and biting responses in the solvent control group lasted for a period of 155.7 ± 10.5 seconds. The time was significantly decreased by pretreatment with FP_MeOH_ (0.1, 0.5 and 1 g/kg) and indomethacin (10 mg/kg) (Figure 3(b)).

### 3.5. Effect of FP_MeOH_ on *λ*-Carrageenan-Induced Mouse Paw Edema

**Figure 4:**
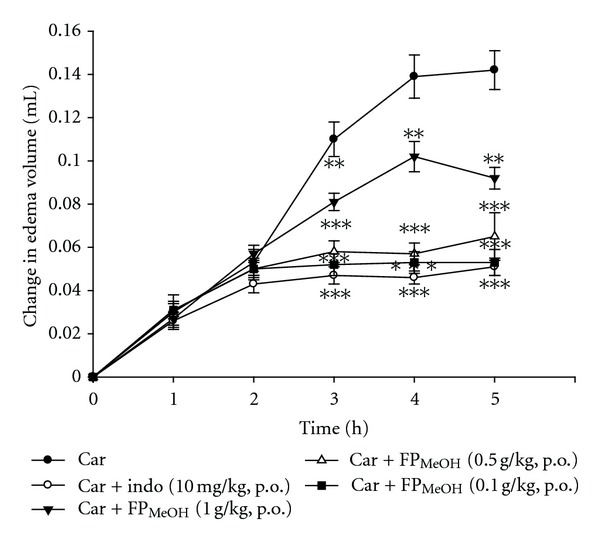
Effects of FP_MeOH_ and indomethacin on *λ*-carrageenan-induced mouse paw edema. The volumes of paw edema were detected at the 1st, 2nd, 3rd, 4th, and 5th h after *λ*-carrageenan injection. Each value represents mean ± SD (*n* = 8). **P < 0.01 and ***P < 0.001 as compared to the *λ*-carrageenan control group (one-way ANOVA followed by Scheffe's multiple range test).

Following the **λ**-carrageenan injection, the volume of mouse paw increased as edema developed, indicating inflammatory activities (Figure 4). However, indomethacin (10 mg/kg) and FP_MeOH_ (0.1–1.0 g/kg) significantly decreased paw edema at the 3rd, 4th, and 5th h after the injection. FP_MeOH_ at the concentrations of 0.5 and 1 g/kg had about equal amount of inhibition as indomethacin.

### 3.6. Histological Analysis

**Figure 5:**
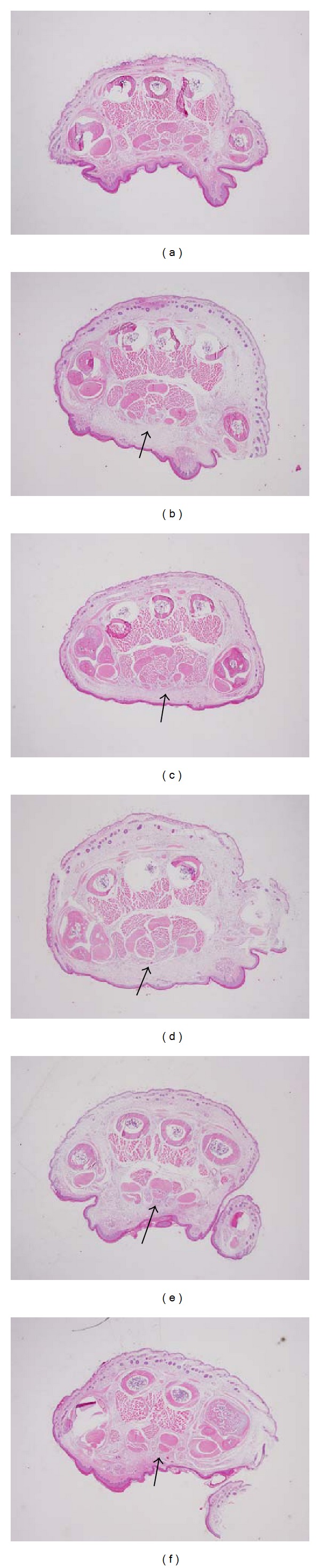
Histopathological examinations on *λ*-carrageenan-induced paw tissue swelling, edema and neutrophil infiltration: (a) normal, (b) *λ*-carrageenan, (c) indomethacin, (d) FP_MeOH_ (0.1 g/kg), (e) FP_MeOH_ (0.5 g/kg), and (f) FP_MeOH_ (1.0 g/kg).

No inflammation, tissue destruction, and swelling phenomenon were observed in the paws of normal mice (Figure 5(a)). On the other hand, the *λ*-carrageenan control group displayed enlarged cavities in the paw tissue (Figure 5(b)). Edematous condition was obviously abated by treatment with 10 mg/kg of indomethacin and 1.0 g/kg of FP_MeOH_ (Figures 5(c) and 5(f)). The severity of mouse paw edema was also graded and summarized in Table 2 (mean ± SD).

### 3.7. Effect of FP_MeOH_ on MDA Level

**Figure 6:**
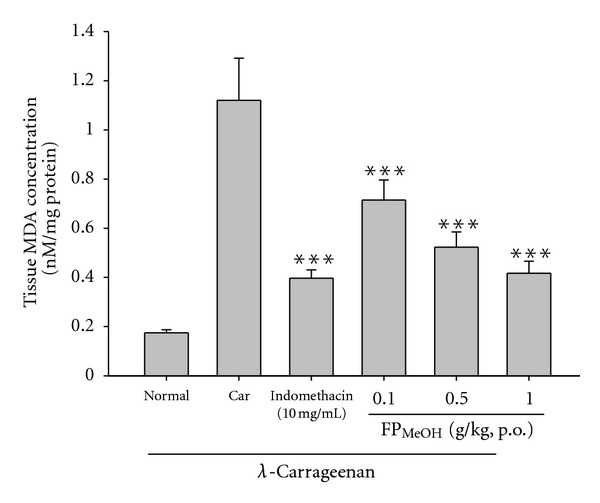
Effects of FP_MeOH_ and indomethacin on MDA concentrations in the edema paws. Each value represents mean ± SD (*n* = 8). ***P < 0.001 as compared to the *λ*-carrageenan control group (one-way ANOVA followed by Scheffe's multiple range test).

MDA level obviously increased in the *λ*-carrageenan control group (1.120 ± 0.172 *μ*M/g); however, pretreatments with FP_MeOH_ (0.1, 0.5 and 1.0 g/kg) and indomethacin (10 mg/kg) significantly inhibited the increase of MDA levels (Figure 6).

### 3.8. Effect of FP_MeOH_ on NO Level

**Figure 7:**
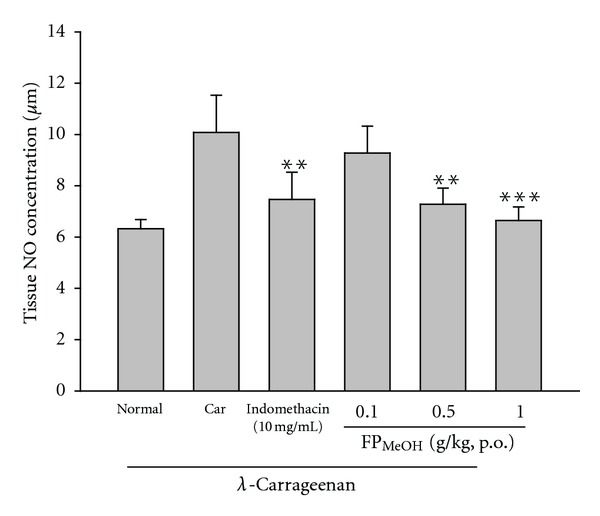
Effects of FP_MeOH_ and indomethacin on NO concentrations in the mouse edema paws. Each value represents mean ± SD (*n* = 8). **P < 0.01 and ***P < 0.001 as compared to the *λ*-carrageenan control group (one-way ANOVA followed by Scheffe's multiple range test).

Pretreatments with FP_MeOH_ (0.5 and 1.0 g/kg) and indomethacin (10 mg/kg) significantly inhibited the increase of NO levels in the edema paws of mice, as compared to the *λ*-carrageenan control group (10.08 ± 1.45 *μ*M), as shown in Figure 7.

### 3.9. Effect of FP_MeOH_ on TNF-*α* and IL-1*β*

**Figure 8:**
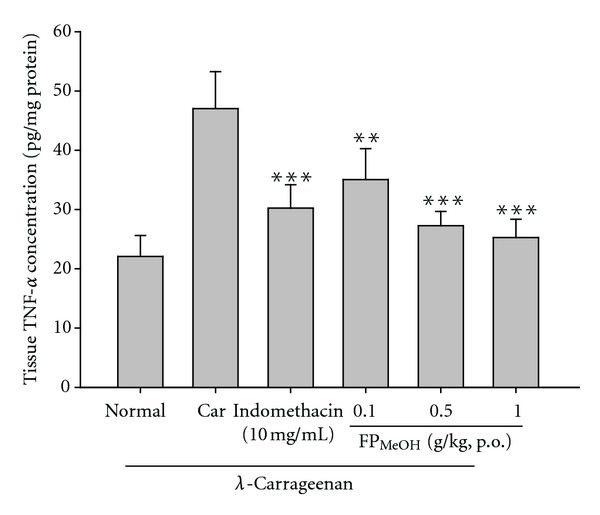
Effects of FP_MeOH_ and indomethacin on TNF-*α* concentrations in mouse paw edema. Each value represents mean ± SD (*n* = 8). **P < 0.01 and ***P < 0.001 as compared to the *λ*-carrageenan control group (one-way ANOVA followed by Scheffe's multiple range test).

**Figure 9:**
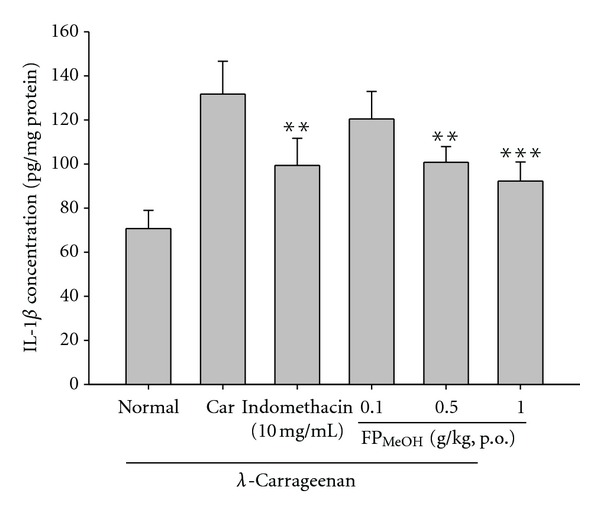
Effects of FP_MeOH_ and indomethacin on IL-1*β* concentrations in mouse paw edema. Each value represents mean ± SD (*n* = 8). **P < 0.01 and ***P < 0.001 as compared to the *λ*-carrageenan control group (one-way ANOVA followed by Scheffe's multiple range test).

 TNF-*α* and IL-1*β* levels in *λ*-carrageenan-induced edema paws were increased remarkably. FP_MeOH_ (0.5 and 1.0 g/kg) and indomethacin (10 mg/kg) significantly reduced the levels of TNF-*α* (Figure 8). Similarly, IL-1*β* levels were significantly lowered by FP_MeOH_ (0.1, 0.5 and 1.0 g/kg) and indomethacin, as shown in Figure 9.

### 3.10. Effect of FP_MeOH_ on COX-2 Level

**Figure 10:**
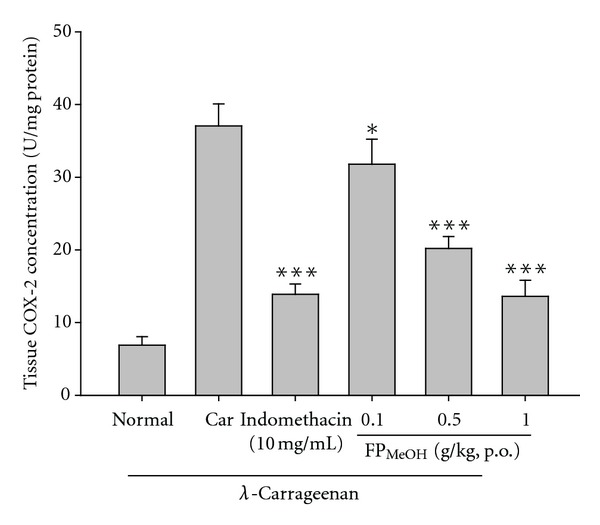
Effects of FP_MeOH_ and indomethacin on COX-2 concentrations in mouse paw edema. Each value represented mean ± SD (*n* = 8). *P < 0.05 and ***P < 0.001 as compared to the *λ*-carrageenan control group (one-way ANOVA followed by Scheffe's multiple range test).

Figure 10 shows that COX-2 level was greatly raised (37.04 ± 3.04 U/mg) in *λ*-carrageenan induced edema paw. However, COX-2 levels were decreased by treating with FP_MeOH_ (0.5 and 1.0 g/kg) as well as indomethacin (10 mg/kg).

### 3.11. Effect of FP_MeOH_ on the Activities of Antioxidant Enzymes

 SOD, GPx, and GRd activities were increased by treating with FP_MeOH_ (0.5 and 1.0 g/kg) and indomethacin (10 mg/kg), as compared to the *λ*-carrageenan control group (Table 1).

## 4. Discussion

*Ficus* is a genus of about 800 species found in tropical and subtropical regions. Several *Ficus* species have been studied for their anti-inflammatory actions, for example, *F. aurantiacea* [20], *F. carica *[21], *F. glomerata* [22], *F. maxima* [23], and *F. obtusifolia* [24].

Traditional medicines have been popularly used in the treatment of various diseases in recent years. Many medicinal plants supply analgesic and anti-inflammatory activities to treat acute, chronic, or recurring illnesses. *Ficus pumila* is a Chinese herbal medicine, its dried stems and leaves have been commonly used in the treatment of rheumatoid arthritis, edema, throat pain, and postpartum abdominal pain [4]. Based on its therapeutic claims in traditional medicine, we investigated possible mechanisms of the analgesic and anti-inflammatory effects of FP_MeOH_.

Analgesic effect of FP_MeOH_ was evaluated by two animal models, including acetic acid-induced writhing response and formalin test. The results of acetic acid induced writhing test showed that FP_MeOH_ (0.1–1 g/kg) and indomethacin (10 mg/kg) provided antinociceptive effects in mice. Acetic acid indirectly triggers the release of nociceptive endogenous mediators (such as bradykinin, serotonin, and prostaglandin) and proinflammatory cytokines (such as TNF-*α* and IL-1*β*) to cause painful sensation [25]. This nociceptive effect can be prevented by analgesic agents with central actions such as morphine as well as peripherally acting drugs like NSAID.

In order to evaluate whether FP_MeOH_ acted centrally or peripherally in the suppression of pain, we also conducted the formalin test. The formalin test involves a biphasic response: the first phase (neurogenic nociceptive response) occurs in the first 5 min after the formalin injection, while the second phase (inflammatory nociceptive response) occurs between 15 to 30 min after formalin injection. Centrally acting drugs can inhibit both phases; however, peripherally acting drugs, such as NSAID, only inhibit the second phase [26]. The treatments of FP_MeOH_ (0.1–1 g/kg) and indomethacin (10 mg/kg) were able to diminish the nociceptive response in the second phase induced by formalin. The results indicated that the antinociceptive effect of FP_MeOH_ was due to its peripheral anti-inflammatory effect.

In the inflammatory model involving **λ**-carrageenan injection, FP_MeOH_ significantly decreased the volume of paw edema. The anti-inflammatory effect was also evident when we compared histopathological examinations of FP_MeOH_-treated groups with that of **λ**-carrageenan control group. Paw biopsy of the **λ**-carrageenan control group showed obvious edematous condition and enlarged cavities in the connective tissues; on the other hand, the groups that received 10 mg/kg of indomethacin or 1 g/kg of FP_MeOH_ had significant improvement in edematous condition and decreased intercellular spaces in connective tissues as shown in Figure 5 and Table 2.

In our follow-up experiments that explored the mechanisms underlying the anti-inflammatory effect of FP_MeOH_, we suspected that suppression of inflammatory cytokines may play an important role. **λ**-Carrageenan-induced paw edema has also been characterized as a biphasic event [13]: histamine, bradykinin, and 5-hydroxytryptamine (5-HT) are released in the first phase of edema (0-1 h), while TNF-*α*, IL-1*β*, COX-2, and PGs are produced more actively in the second phase (1–6 h). TNF-*α* and IL-1*β* have been reported in several studies to be able to recruit leukocytes, such as neutrophils [27, 28]. Additionally, COX-2 is an enzyme responsible for increasing prostaglandin levels in inflammatory reactions, which could in turn worsen the degree of swelling [29]. In this study, FP_MeOH_ (dose dependent) and indomethacin (10 mg/kg) showed significant anti-inflammatory effect in **λ**-carrageenan-induced mouse paw edema from the 3rd to 5th h. Moreover, the levels of TNF-*α*, IL-1*β*, and COX-2 were also decreased by treating with FP_MeOH_ and indomethacin (10 mg/kg). Thus, a putative anti-inflammatory mechanism of FP_MeOH_ could be associated with the inhibition of inflammatory mediators, such as TNF-*α*, IL-1*β*, and COX-2.

Another possible mechanism behind the anti-inflammatory effect of FP_MeOH_ could be due to its antioxidative activity. Increased production of TNF-*α*, IL-1*β*, and other proinflammatory cytokines not only has the capacity to recruit leukocytes, but would also trigger their release of active substances such as ROS [30]. Nitric oxide, hydrogen peroxide, hydroxyl radicals, and superoxide anions play major roles in terms of producing cellular damage [31]. MDA is a reactive aldehyde caused by toxic stress in cells and form covalent protein adducts which are referred to as advanced lipid peroxidation end products (ALE). Inflammation would result in the accumulation of MDA; on the other hand, enhancing the level of GPx, GRd and SOD could reduce MDA production [32]. In this study, SOD, GPx, and GRd activities increased in the liver after treatment with FP_MeOH_. Conversely, the level of MDA decreased significantly. Therefore, we believe that the suppression of MDA production was likely associated to the increase in SOD, GPx, and GRd activities.

The fingerprint chromatogram of FP_MeOH_ was established, and its three flavonoid contents (rutin, luteolin and apigenin) were quantitatively determined. Three active compounds of FP_MeOH_, rutin, luteolin, and apigenin, have been previously demonstrated to possess anti-inflammatory and antinociceptive activities which may be responsible, at least in part, to the anti-inflammatory and antinociceptive effects of FP_MeOH_ [5–7].

This study demonstrated that *Ficus pumila* exhibited antinociceptive and anti-inflammatory activities. The anti-inflammatory mechanisms of FP_MeOH_ against **λ**-carrageenan-induced paw edema involved two possible pathways. The first pathway alleviated the levels of inflammatory factors, such as IL-1*β*, TNF-**α*,* and COX-2 in the edema paw induced by **λ**-carrageenan. The other pathway was likely associated to the decrease in MDA and NO levels in the edema paw via increasing the activities of SOD, GPx, and GRd in the liver. In conclusion, this study supported possible mechanisms of FP_MeOH_ used for mitigating inflammatory pain.

## Figures and Tables

**Table 1:** Effect of FP_MeOH_ and indomethacin on liver SOD, GPx, and GRd activities in mice injected with **λ**-carrageenan.

Groups	SOD (U/g protein)	GPx (U/mg protein)	GRd (U/mg protein)
Normal	121.45 ± 12.53	1.579 ± 0.136	0.106 ± 0.012
Car	67.69 ± 16.43	0.949 ± 0.144	0.063 ± 0.014
Car + Indomethacin	99.72 ± 10.36**	1.222 ± 0.109	0.085 ± 0.006**
Car + FP_MeOH_ (0.1 g/kg)	84.94 ± 13.82*	1.287 ± 0.133*	0.082 ± 0.008**
Car + FP_MeOH_ (0.5 g/kg)	93.89 ± 16.68*	1.332 ± 0.207**	0.085 ± 0.007**
Car + FP_MeOH_ (1.0 g/kg)	95.48 ± 17.87*	1.339 ± 0.224**	0.078 ± 0.006*

Each value represents the mean ± SD (*n* = 8). *P < 0.05 and **P < 0.01 as compared with the *λ*-carrageenan (Car) group (one-way ANOVA followed by Scheffe's multiple range test).

**Table 2:** Effects of FP_MeOH_ and indomethacin on the severity of mouse paw edema.

Groups	Grade (mean ± SD)
Car	3.6 ± 0.55
Car + Indomethacin	2.0 ± 0.71*
Car + FP_MeOH_ (0.1 g/kg)	3.6 ± 0.55
Car + FP_MeOH_ (0.5 g/kg)	3.0 ± 0.00
Car + FP_MeOH_ (1.0 g/kg)	2.2 ± 0.45*

Each value represents the mean ± SD (*n* = 5). *P < 0.05 as compared with the *λ*-carrageenan (Car) group (one-way ANOVA followed by Scheffe's multiple range test).
